# Accuracy of teledentistry versus clinical oral examination for aged-care home residents: A pilot study

**DOI:** 10.1016/j.tjfa.2024.100001

**Published:** 2025-01-01

**Authors:** Mennatollah Nagy Sharkawy, Maii Mohamed, Hala M. Abbas

**Affiliations:** a Department of Pediatric Dentistry and Dental Public Health, Faculty of Dentistry, Cairo University, Cairo, Egypt; b Department of Pediatric Dentistry and Dental Public Health, School of Dentistry, Newgiza University, Giza, Egypt

**Keywords:** Teledentistry, Mhealth, Geriatric dentistry, Digital health, Dental triaging

## Abstract

**Objectives:**

The aim of this pilot study is to assess the feasibility of using the mobile photographic method (m-health) of teledentistry for oral screening and triaging of older patients living in aged care homes.

**Methods:**

The study took place in 2023 in four aged care facilities in Egypt. Three trained dentists performed clinical oral examinations of the participants and filled in the World Health Organization (WHO) oral health assessment form. Afterwards, five intraoral photographs were taken for each participant and independently examined by three calibrated dentists for caries detection and decision on intervention urgency for proper dental referral. Agreement between the testing modalities was analyzed using Cohen's kappa coefficient, and the significance level was set at *p* < 0.05 within all tests.

**Results:**

The results indicated that teledentistry had higher specificity than sensitivity in caries detection compared to clinical examination. The level of agreement between the teledentistry examination and the clinical oral examination for caries assessment ranged from (*k* = 0.36) to (*k* = 0.58) showing fair to moderate agreement. Also, all teledentistry examiners showed almost perfect statistically significant intra-rater and inter-rater agreement for caries detection (*K* ≥ 0.82, *p* < 0.001). Moreover, intervention urgency scoring showed moderate to substantial agreement between the testing modalities with kappa values ranging from (*k* = 0.48) to (*k* = 0.65).

**Conclusions:**

The mobile photographic method of teledentistry offers a feasible model that helps in oral examination and triaging dental treatment of older patients living in aged care facilities. However, larger studies with proper sample size are required which will allow better generalizability of the results.

## 1 Introduction

Oral health is an integral component of general health and plays an important role in the individuals’ quality of life [1]. As the global population is aging, the number of adults aged 65 and older reached 761 million (9.6 %) in 2021 and it is expected to reach 1.6 billion people (17 %) by 2050 [2]. This increasing number of the older population is associated with more suffering from untreated oral and dental diseases due to the low level of oral care provision. Therefore, improving the older adult's accessibility to oral care has become crucial [3]. In addition, the low accessibility of the older adults to oral care was amplified by the coronavirus (SARS-CoV-2) pandemic which has affected public health systems around the world [4]. The coronavirus has also limited dental care to urgent treatments due to the high risk of spread of infection during dental visits [[5], [6], [7]]. Thus, telemedicine was introduced to mitigate the older people's rising challenges of low accessibility to healthcare facilities, high costs of dental treatment and the need to amplify the reach of dental healthcare professionals [[8], [9], [10]].

Teledentistry is the branch of telemedicine which is concerned with dental and oral health. It uses information technology to share the patients’ images and relevant information for diagnostic purposes with dental health professionals [[11], [12], [13]]. Therefore, teledentistry uses telecommunication to facilitate the accessibility of high-risk populations to oral care, reduce the inequalities they face in dental care, and reduce the extra costs of dental visits [14,15]. Thus, teledentistry is considered an acceptable method of dental examination among patients and their caregivers [16].

Older people living in aged care homes are highly vulnerable to oral and dental diseases [[17], [18], [19]]. Their reduced mobility, combined with their increased risk of infections, prevents them from receiving conventional treatment in dental clinics. Therefore, triaging dental visits through teledentistry in residential aged-care facilities will improve the accessibility to oral care and reduce the transportation inconveniences [16,20]. This can be facilitated using mobile health (mHealth) with smartphones-obtained photographs. mHealth has shown an acceptable diagnostic level in both dental caries detection and oral cancer screening, making it a valid method for examining older patients [14,15].

To the best of our knowledge, no previous studies assessing the accuracy of teledentistry among older adults living in aged-care homes in Egypt have been conducted. This pilot study aims to assess the feasibility of using the mobile photographic method (mHealth) of teledentistry for oral screening and triaging of older patients in aged care homes. This is assessed by measuring the diagnostic accuracy of the mobile photographic method versus clinical oral examination for the same group of participants.

## 2 Methods

This study is an observational cross sectional pilot study that took place in Egypt. The study was conducted under ethical approval from the Ethics Committee, with approval number 10/11/22, at the School of Dentistry, Cairo University, Egypt. The protocol of the study is registered with ClinicalTrials.gov Identifier: NCT05101681. Additionally, the methods and the results are reported according to the Standards for Reporting of Diagnostic Accuracy (STARD) statement [21].

### 2.1 Study settings

The study took place from January to March 2023 in four aged-care homes in Egypt. The care homes were selected conveniently; three were located in Giza Governorate and one in Cairo Governorate, where no regular oral care was provided for the older adults. However, occasionally some non-governmental organizations do visits and provide dental treatment for the residents who needed it.

### 2.2 Participants and sampling

Purposive sampling was used to recruit the study sample. Since no sample size calculation is required for a pilot study, 30 older adults were recruited as a reasonable sample size [22,23]. The participants recruited were residents of the selected aged care homes. They were also recruited based on their capacity to effectively communicate and provide informed consent for their examination. The residents were excluded from the study if they were unwilling to participate in the study or if they had a mental disorder that prevented them from communicating or giving informed consent. This information was obtained from the facility managers of the aged-care homes based on the residents’ medical records.

Additionally, research indicates that oral health education enhances positive oral health behavior among the older population [24]. Thus, before the oral examination, the residents attended a session where examiners used posters to demonstrate the importance of a healthy diet, oral hygiene, and early diagnosis and treatment. The session also included demonstrations of teeth brushing, dental flossing, denture cleaning, and self-examination for early signs of oral cancer.

Finally, the examiners informed the residents about the aim of the research and explained the oral examination process. The eligible older adult residents who were willing to participate signed an informed consent which included the aim and the description of the research.

### 2.3 Clinical oral examination

The clinical oral examination was performed at the aged-care homes by three dentists with at least five years of experience and were candidates of the Dental Public Health master's program at Cairo University. The dentists were calibrated to use the scoring system of the World Health Organization (WHO) oral health assessment form for adults for the dental examination and intervention urgency assessment. This form follows the guidelines for oral health surveys (Annex). Before examining the residents, the dentists performed examinations of 10 older patients using the WHO form as a form of self-calibration. The level of agreement between the examiners ranged from (*k* = 0.94) to (*k* = 0.96), showing an almost perfect agreement [25].

The caries assessment was done at the tooth level following the WHO protocol [26]. The photographs provide a two-dimensional image; therefore, it is impossible to perform assessment of all tooth surfaces. Thus, only occlusal surfaces of teeth were assessed for presence or absence of caries. The crown was assigned a score of 0 if it was considered sound and a score of 1 if it was considered carious. A sound crown must not exhibit any signs of treated or untreated caries, and it had to be cavitated to be classified as carious. Also, a crown was considered sound if it showed white or chalky spots, pre-cavitation lesions, or stained pits and fissures. Lesions that were not of carious origin, such as abrasion and enamel fluorosis, were not classified as caries. Conversely, a tooth with a temporary filling was classified as carious. Moreover, interproximal, root caries, missing and filled teeth were excluded from the examination due to the difficulty of detecting these lesions using photographs. This exclusion was necessary to accurately measure diagnostic accuracy and agreement based on the presence or absence of dental caries. Notably, no radiographs were taken during this assessment.

The intervention urgency scores were based on the basic methods of oral surveys published by the WHO [26].The scores range from 0 to 4 where 0 means no intervention is required and 1 means preventive or routine care are required .Score 2 means a timely treatment is needed such as dental caries management and scaling, whereas score 3 means an immediate treatment is necessary due to pain or infection. Finally score 4 means that an appropriate referral for specialized medical or dental treatment is required due to a systemic condition or oral lesion [26].

Adhering to the infection control measures, disposable diagnostic kits and napkins were used for the oral examination of the participants. Each disposable diagnostic kit consisted of a disposable mirror, a disposable dental probe, and a disposable tweezer. The participants were examined in a well lightened room, where some participants were seated while others were lying on their beds. The three examiners performed the examination independently and filled in one WHO form for every participant. Each participant had a unique identification number to be used later for the teledentistry examination while keeping the patients anonymous. Additionally, the examiners recorded the participants’ demographic information. Upon examination, the findings were recorded in the dentition status of permanent teeth and the intervention urgency sections.

### 2.4 Intraoral photographs acquisition

The participants remained in the same examination location, where a trained dental student took the intraoral photographs of each participant using a smartphone camera. The dental student received training to capture intraoral images of different dental views (frontal, right lateral, left lateral, upper occlusal, and lower occlusal) with good quality for diagnosis. The images must depict the teeth from all angles, without any physical obstructions or flares that could impede a clear visualization of the surface for diagnosis purposes. During the training process, the dental student practiced taking these photographs on volunteers using the same mobile phone that was used in the study.

Disposable cheek retractors were used to aid in better vision of the participant's oral cavity. No mirrors were used for taking the intraoral photographs. A smartphone camera (Samsung Galaxy Note 9®) was used using autofocus and automated settings. Only room lighting and the flashlight of the mobile phone camera were used. For consistency, the participants must bite posteriorly in normal occlusion while capturing the frontal views for clear visibility of the upper and lower teeth and the supporting periodontium. The focus was set at the most anterior teeth and the gingival margins of the teeth must be visible in both the frontal and lateral views. In the occlusal views, the participants were instructed to have a maximum mouth opening for better visibility of the occlusal teeth surfaces.

**Fig. 1:**
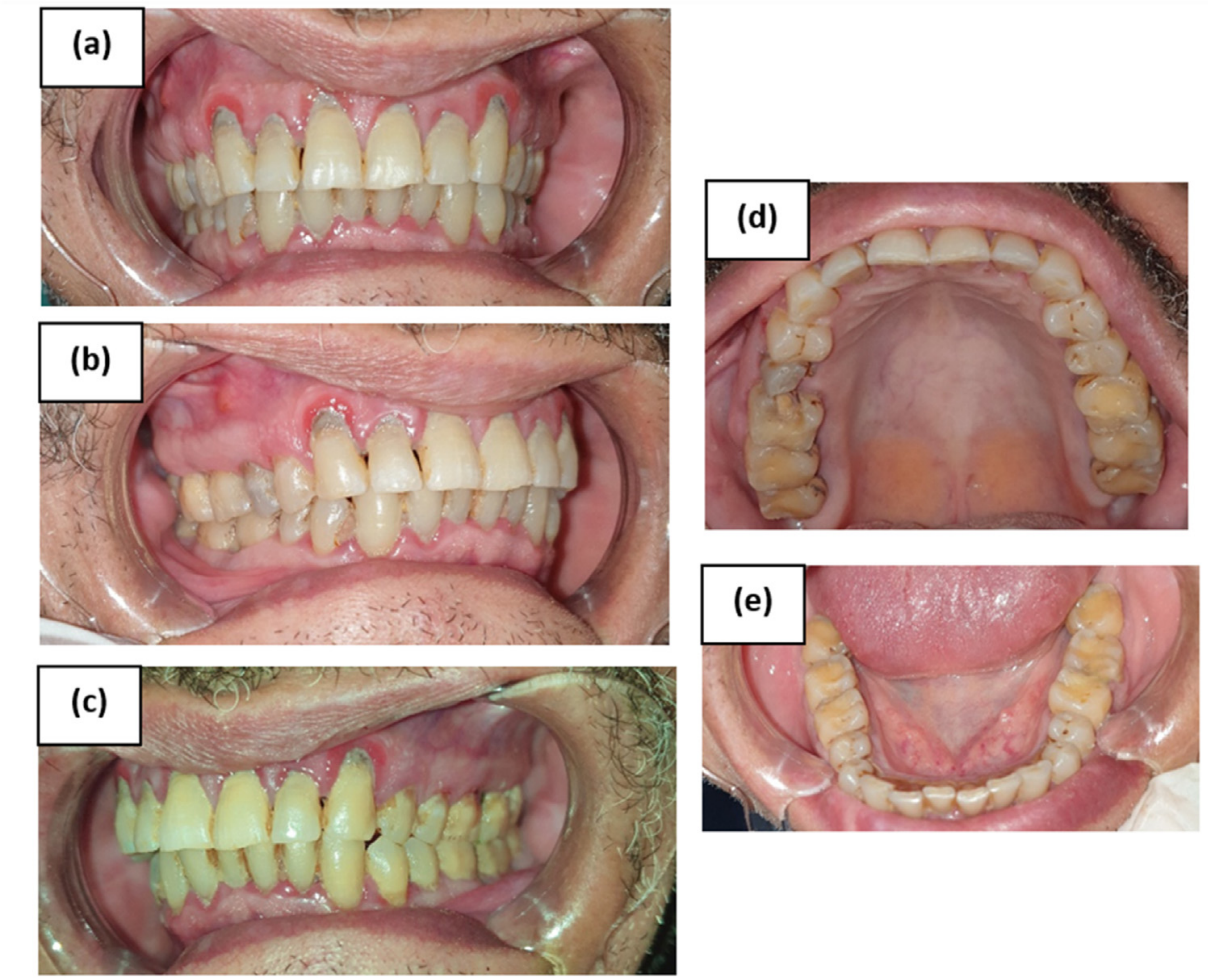
Sample of intraoral photographs showing 5 dental views: (a)Frontal view, (b)Right lateral view, (c)Left lateral view, (d)Upper occlusal view and (e) Lower occlusal view.

The dental student took five intraoral photographs for each participant, which consisted of frontal view, right lateral view, left lateral view, upper occlusal view, and lower occlusal view (Fig. 1). The photographs included only the patients’ dentition while the patients remained anonymous. The photographs were stored directly on the mobile phone in separate folders. Each folder had a unique number that was given by the examiners to each participant. Subsequently, the 30 created folders were then uploaded on google drive which is a service developed by google that allows storage and sharing of different files in the cloud. The teledental examiners used the uploaded folders for the subsequent examination.

### 2.5 Teledentistry photographic assessment

The teledentistry group consisted of three dentists with at least five years of experience, different from the dentists who performed the clinical oral examination. The three dentists were calibrated and trained by an independent dental examiner to fill the WHO form using the patients’ intraoral photographs. The level of agreement between the three teledentistry examiners and the gold standard ranged from moderate to substantial, (with kappa values of 0.58, 0.46, and 0.75 for examiners 1, 2, and 3, respectively. The teledentistry examiners received six hours of training. The first 2-hour training session was on how to fill out the Excel sheets using the WHO form. During the following 4-hour session, they received training on the examination of the images of a random sample of 10 intraoral photographs as suggested by the WHO [26].The training involved both caries assessment and intervention urgency scoring in accordance with the WHO criteria. The images used during the training were obtained from patients who were not part of the study sample. The teledentistry examiners were blinded to the scores of the gold standard clinical oral examination.

The link to google drive folders that were previously created was shared with the three dentists to examine the pictures of each patient anonymously. The teledentistry group recorded their scores independently in Excel sheets conforming to the dentition status of permanent teeth, and the intervention urgency sections.

Each teledentistry examiner then sent their Excel sheets to be compared with the gold standard assessment. The biostatistician was blinded to the gold standard and teledentistry examination pictures.

Finally, the participants who needed dental intervention were referred to the university's teaching dental hospital to receive dental treatment. The referrals were based on the intervention urgency scores from the WHO Basic Oral Surveys [26], which were assigned to each participant.

### 2.6 Statistical analysis

Categorical data were presented as frequency and percentage values, while numerical data were presented as mean and standard deviation values. The mode of the scores given by the three clinical examiners was included in all analyses. Agreement between the testing modalities and the repeated observations was analyzed using Cohen's kappa coefficient [25]. Sensitivity, specificity, positive predictive value, negative predictive value, and accuracy were calculated for each teledentistry examiner. The significance level was set at *p* < 0.05 for all tests. Statistical analyses were conducted using R statistical analysis software version 4.3.1 for Windows.^1^ To calculate the intra-examiner agreement, the sample was recharted by the teledentistry examiners after a wash out period of at least 4 weeks after the initial assessment.

## 3 Results

### 3.1 Demographic data of the study participants

The recruited sample comprised 28 women and only 2 men, as two of the aged-care homes had only female residents. The mean age of the participants was (73.20±8.43) (Mean±SD) years. Most of the participants had not received any form of education (36.7 %), while nearly quarter of the sample received higher education (26.7 %).

### 3.2 Caries prevalence and intervention urgency scores

Upon clinical oral examination, the caries prevalence among older adults was 14.7 % of the examined teeth (*n* = 455) across the 30 participants. This differed from the assessments of the three teledentistry examiners as shown in Table (1). Where caries prevalence ranged from as low as 11.6 % (third examiner) to as high as 25.5 % (second examiner).

**Table 1:** Caries prevalence as assessed by clinical oral examination and teledentistry examiners.

Caries prevalence	Number (n)	Percentage (%)	p-value
Clinical oral examination	67	14.7	<0.001*
Teledentistry examiner 1	74	16.3	
Teledentistry examiner 2	116	25.5	
Teledentistry examiner 3	53	53	

⁎ significant (*p* < 0.05).

The intervention urgency scoring for the two examination modalities is presented in Table (2). The clinical oral examination indicated that 14 patients required prompt treatment and another 14 required immediate treatment. Additionally, only one patient was identified for preventive treatment, and one was referred for comprehensive evaluation. Although the first teledentistry examiner assigned the same number of patients to each category of intervention urgency, this does not reflect 100 % agreement with the clinical oral examination.

**Table 2:** Intervention urgency scores as assessed by clinical oral examination and teledentistry examiners.

Level of urgency	Preventive or routine treatment n (%)	Prompt treatment	Immediate treatment needed	Referred for comprehensive evaluation	p-value
Clinical oral examination	1 (3.3)	14 (46.7)	14 (46.7)	1 (3.3)	<0.001*
Teledentistry examiner 1	1 (3.3)	14 (46.7)	14 (46.7)	1 (3.3)	
Teledentistry examiner 2	0 (0)	8 (26.7)	21 (70)	1 (3.3)	
Teledentistry examiner 3	2 (6.7)	5 (16.7)	23 (76.7)	0 (0)	

⁎ significant (*p* < 0.05).

## 3.3 Diagnostic accuracy

### 3.3.1 Caries assessment

The diagnostic accuracy metrics between the teledentistry examiners and the gold standard (clinical oral examination) are shown in Table (3). The teledentistry examiners performed caries assessment for a total of 455 teeth. Using a 95 % confidence interval (CI), the sensitivity of caries assessment by teledentistry examination compared to the gold standard ranged from 56.7 % to 65.7 %. The specificity ranged from 81.4 % to 96.1 %. The results showed a higher negative predictive value (NPV) than positive predictive value (PPV). The overall diagnostic accuracy ranges from 79.1 % to 90.3 %.

**Table 3:** Diagnostic performance and inter-examiner agreement between teledentistry and clinical oral examination for dental caries assessment among older adults.

Parameter (%)	Teledentistry examiner 1 Vs Gold standard	Teledentistry examiner 2 Vs Gold standard	Teledentistry examiner 3 Vs Gold standard
Sensitivity (95 % CI)	59.7 % (48 %−71.5 %)	65.7 % (54.3 %−77 %)	56.7 % (44.9 %−68.6 %)
Specificity (95 % CI)	91.2 % (88.4 %−94 %)	81.4 % (77.6 %−85.3 %)	96.1 % (94.2 %−98 %)
PPV (95 % CI)	54 % (42.7 %−65.4 %)	37.9 % (29.1 %−46.8 %)	71.7 % (57.7 %−83.2 %)
NPV (95 % CI)	92.9 % (90.3 %−95.5 %)	93.2 % (90.5 %−95.9 %)	92.8 % (89.8 %−95.1 %)
Accuracy (95 % CI)	86.6 % (83.5 %−89.7 %)	79.1 % (75.4 %−82.9 %)	90.3 % (87.6 %−93 %)
Cohen's Kappa (95 % CI)	0.49 (0.37–0.61) <0.001*	0.36 (0.25:0.48) <0.001*	0.58 (0.46–0.70) <0.001*

Examiner=teledentistry examiner; Gold standard =clinical oral examination.

PPV=positive predictive value, NPV=negative predictive value, CI= confidence interval.

⁎ Significant (*p* < 0.05).

## 3.4 Reliability and agreement of teledental examinations

### 3.4.1 Inter-examiner reliability

#### 3.4.1.1 Caries assessment

The inter-examiner reliability between the teledentistry examiners and clinical examination ranged from (*k* = 0.36) to (*k* = 0.58) for caries assessment which showed a fair to moderate agreement, Table (3). In addition, the inter-observer reliability between the three teledentistry examiners for caries assessment was substantial with Fleiss’ Kappa of 0.76 (κ = 0.76, *p* < 0.001).

#### 3.4.1.2 Intervention urgency

The level of agreement between the teledentistry examiners and the clinical oral examination for intervention urgency scoring ranged from moderate to substantial agreement as shown in Table (4). Additionally, the percentage of agreement between both examination modalities ranged from 60 % to 73.3 % which is presented in Table (4). Also, the inter-examiner agreement between the three teledentistry examiners was almost perfect (κ = 0.83, *p* < 0.001).

**Table 4:** Inter-examiner agreement and percentage of agreement between teledentistry and clinical oral examination for intervention urgency scoring among older patients.

Parameter	Teledentistry examiner 1 Vs Gold standard	Teledentistry examiner 2 Vs Gold standard	Teledentistry examiner 3 Vs Gold standard
Cohen's Kappa (95 % CI)	0.65 (0.09:1.00) <0.001*	0.62 (0.19:1.00) <0.001*	0.48 (0.45:1.00) <0.001*
Percentage of agreement	73.3 %	73.3 %	60 %

Examiner=teledentistry examiner; Gold standard =clinical oral examination.

CI= confidence interval.

⁎ Significant (*p* < 0.05).

### 3.4.2 Intra-examiner reliability

Intra-examiner agreement is presented in Table (5), for all observers there was an almost perfect statistically significant agreement between both observations for caries assessment (*k* > 0.82, *p* < 0.001). For intervention urgency, the intra-examiner agreement among the three teledentistry examiners for both the original and the duplicate examinations showed an almost perfect agreement (*k* ≥ 0.82, *p* < 0.001).

**Table 5:** Intra-examiner agreement among teledentistry examiners for dental caries assessment and intervention urgency scoring among older patients.

Examiner	Cohen's kappa (95 % CI)		p-value
	Dental Caries	Intervention Urgency	
First	0.97 (0.95:0.98)	0.82 (0.5:1.0)	<0.001*
Second	0.82 (0.79:0.86)	1.0 (0.7:1.0)	
Third	0.97 (0.95:0.98)	0.91 (0.6:1.0)	

CI= confidence interval.

⁎ Significant (*p* < 0.05).

## 4 Discussion

This study aimed to assess the feasibility of using teledentistry for caries detection and dental triaging among older adults in aged-care facilities. The results showed that teledentistry had higher sensitivity than specificity for caries detection. Agreement between teledentistry and clinical examination for caries detection ranged from fair (*k* = 0.36) to moderate (*k* = 0.58). For intervention urgency scoring, the agreement ranged from moderate (*k* = 0.48) to substantial (*k* = 0.65). Although the sample size is not large enough to be representative of the whole population, the study underscores the potential of using teledentistry in aged-care homes.

Teledentistry acts as a model of communication between primary health care providers and specialists leading to fewer referrals and efficient use of resources [27],therefore teledentistry is beneficial for the vulnerable populations. The integration between oral health education, teledentistry and dental referral was found to be effective in improving the oral health outcomes of older adults in residential aged care facilities[28] .Consequently, our study adopted the same approach and implemented the same three elements. Unlike other studies that used intraoral cameras for teledentistry, our study employed a smartphone camera. This approach was chosen due to its affordability and accessibility to a broader population. We also used the asynchronous teledentistry model recognized as the most affordable model of teledentistry in residential aged care homes [29].This model also allows flexibility for the dentists to review the images at their convenience.

The sensitivity of teledentistry for caries detection was lower than the specificity for all three teledentistry examiners. This high specificity level means a lower chance of false positive results (recording a tooth as carious when it is sound). This result is consistent with another study that suggested low sensitivity values are due to the exclusion of missing and filled teeth [30], which have a high probability of being identified on the photographs [31]**.** Therefore, there are several missing true positive findings unlike the true negative scores.

A study on a teledental application reported a sensitivity of 57 %, comparable to the current study's sensitivity range of (56.7 %−65.7 %), while the specificity reported was 100 %, aligning with the high specificity observed in our study [31]. Conversely, another study concluded that the sensitivity and kappa scores of caries detection in permanent teeth are lower than primary teeth [32]**.** This explains the low sensitivity and kappa levels in our study compared to studies where examiners examined primary teeth [32,33].The difference in the levels of sensitivity between the teledentistry examiners in our study can be attributed to the higher competence of one over another to detect carious lesions. Also, it can be attributed to the tendency of the examiner to record the tooth as carious when it is not clear on the photograph if it is sound or carious.

The results also revealed that there is moderate agreement between the first and third teledentistry examiners and clinical examination for caries detection, these results are much the same as [30,31,34] but lower than [32,33]. However, the second examiner showed fair agreement with clinical examination which shows that there is variability between the dentists in the clinical decisions. The variation in the level of agreement between the examiners and clinical examination in the current study can be due to the confusion between staining and caries and the difference in fissures morphology of posterior teeth [35,36]**.**

Also, the inter-examiner reliability for caries detection among the three teledental examiners showed substantial agreement (κ = 0.76) which is higher than other teledentistry studies with kappa values of 0.61, 0.62 and 0.68 respectively [30,31,34]. Similarly, the intra-examiner reliability of the teledentistry examiners was almost perfect for all three examiners, which implies high consistency in the examination. This is consistent with another study that used the mobile photographic dental screening of school children and showed an intra-examiner reliability ranging from substantial to almost perfect agreement [32].

Triaging patients using teledentistry is recommended by several studies especially for high-risk groups like the older adults as it is an essential part of their referral system [28,[37], [38], [39]].To the best of our knowledge, no other studies have used the intervention urgency scoring section in the WHO form. Our results showed a moderate to substantial level of agreement between teledentistry and clinical examination in the intervention urgency scoring for dental triaging of older patients. As a result, the older patients were referred to the university's hospital to receive the necessary dental treatment based on the urgency scores determined during their triaging. Those requiring a comprehensive evaluation (Score 4) were referred first, followed by those needing immediate treatment (Score 3). Finally, patients who required prompt and preventive treatment (Scores 2 and 1, respectively) were referred last.

Our study has the limitation of being a pilot study which cannot be used for hypothesis testing. Also, the results of a pilot study cannot be generalized to the whole population which creates a limitation in the study's external validity [40]**.**Additionally, purposive samples have the advantage of a homogeneous sample of interest. However, purposive samples imply the limitation of external validity as the results cannot be generalized except to a similar population [41]. Another limitation is that our study sample includes only 2 men and 28 women, which is not representative of the whole population, limiting the generalizability of the results. Also, the photographic images are two-dimensional, which limits the detection of interproximal and root caries; therefore, these were excluded from our study [34]. Additionally, since photographic images are more accurate in the detection of dental pathologies on smaller areas [30], using full-mouth images may have reduced the accuracy of the results**.**

## 5 Conclusions

The mobile photographic method of teledentistry offers a feasible and affordable model for caries detection and triaging the dental treatment for older adults living in aged-care homes. Therefore, raising awareness about the role and importance of teledentistry among the dental stakeholders is vital.

### 6 Recommendations and implications for future research

Larger studies with proper sample sizes are required to allow better generalizability of the results.

It is also recommended to advocate for geriatric dentistry to be recognized as a specialty to serve the growing older population, as it's not yet in many countries [42]. Additionally, artificial intelligence (AI) and teledentistry could be integrated in the future. This would enhance data processing and improve dental care quality [43]. However, attention must be given to the necessary infrastructure for the technology and to data privacy.

## Declaration of generative AI and AI-assisted technologies in the writing process

No generative AI or AI-assisted technologies have been used in the writing process.

## CRediT authorship contribution statement

**Mennatollah Nagy Sharkawy:** Conceptualization, Data curation, Investigation, Methodology, Project administration, Resources, Validation, Writing – original draft. **Maii Mohamed:** Supervision, Writing – review & editing. **Hala M. Abbas:** Supervision.

## Declaration of competing interest

The authors declare no conflict of interest for this research. Also, the authors received no financial support for this research.

## Figures and Tables

**Figure:**
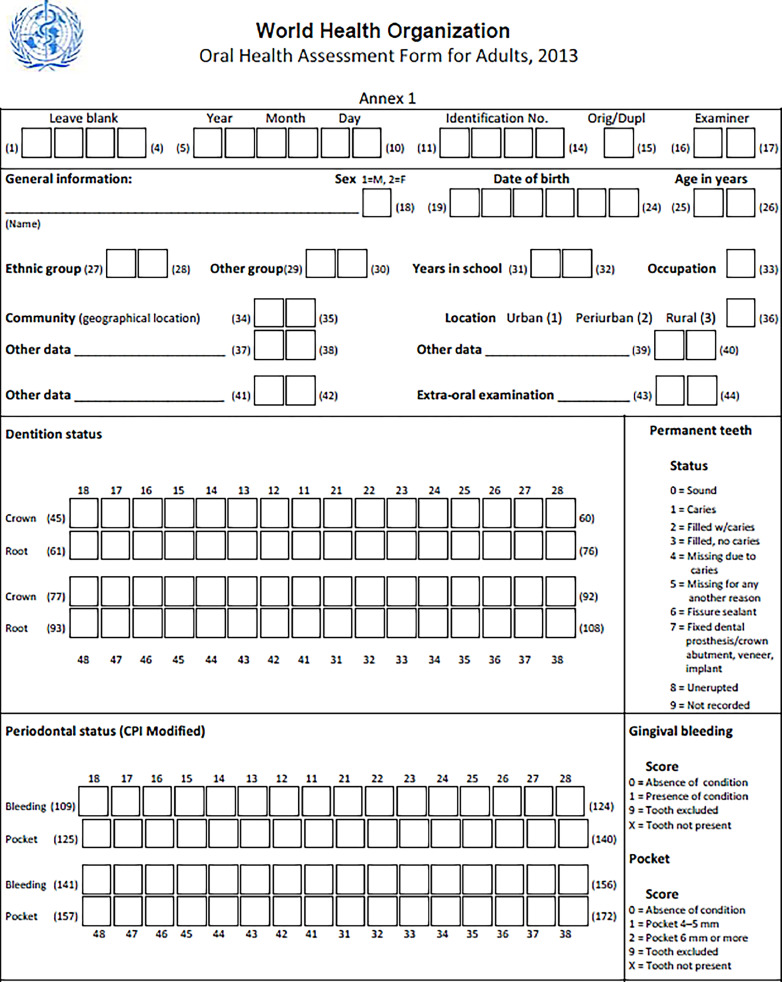


**Figure:**
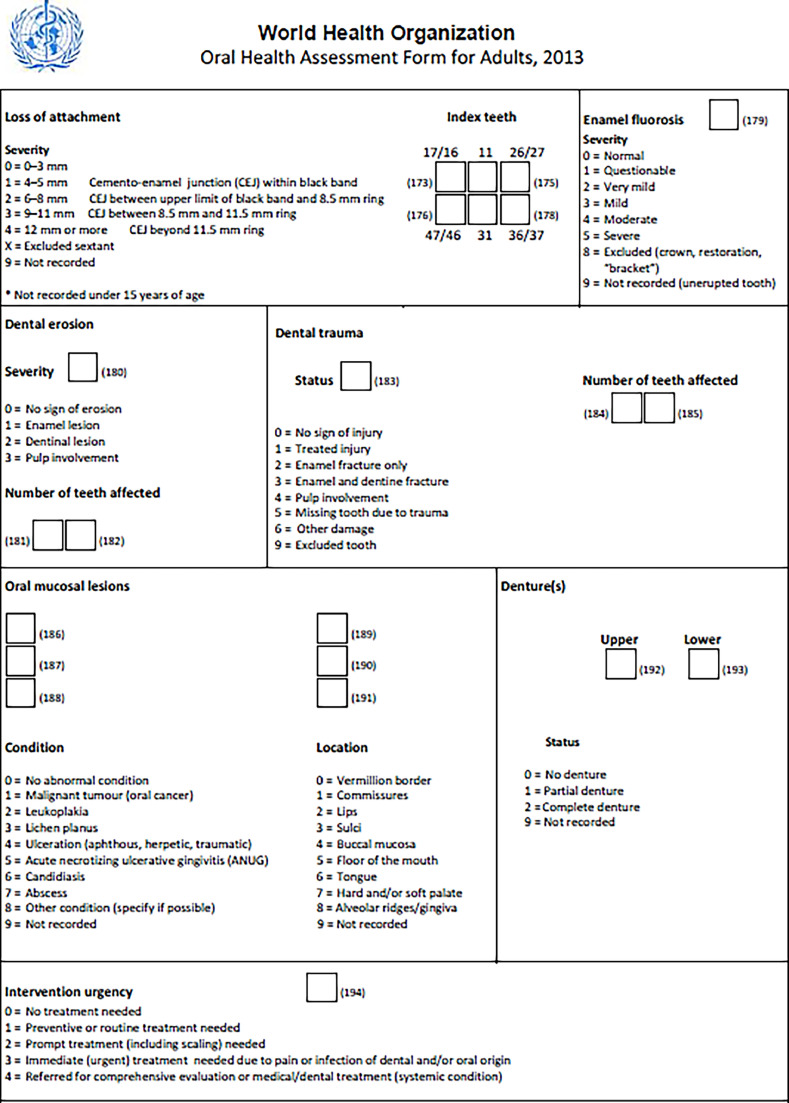


## Footnotes

1 R Core Team (2023). R: A language and environment for statistical computing. R Foundation for Statistical Computing, Vienna, Austria. URL https://www.R-project.org/.
